# Competing Endogenous RNAs, Non-Coding RNAs and Diseases: An Intertwined Story

**DOI:** 10.3390/cells9071574

**Published:** 2020-06-28

**Authors:** Ugo Ala

**Affiliations:** Department of Veterinary Sciences, University of Turin, 10124 Turin, Italy; ugo.ala@unito.it

**Keywords:** competing endogenous RNAs, ceRNA mechanism, miRNA, non-coding RNAs, cancer, cardiovascular pathologies, neurodegenerative disorders

## Abstract

MicroRNAs (miRNAs), a class of small non-coding RNA molecules, are responsible for RNA silencing and post-transcriptional regulation of gene expression. They can mediate a fine-tuned crosstalk among coding and non-coding RNA molecules sharing miRNA response elements (MREs). In a suitable environment, both coding and non-coding RNA molecules can be targeted by the same miRNAs and can indirectly regulate each other by competing for them. These RNAs, otherwise known as competing endogenous RNAs (ceRNAs), lead to an additional post-transcriptional regulatory layer, where non-coding RNAs can find new significance. The miRNA-mediated interplay among different types of RNA molecules has been observed in many different contexts. The analyses of ceRNA networks in cancer and other pathologies, as well as in other physiological conditions, provide new opportunities for interpreting *omics* data for the field of personalized medicine. The development of novel computational tools, providing putative predictions of ceRNA interactions, is a rapidly growing field of interest. In this review, I discuss and present the current knowledge of the ceRNA mechanism and its implications in a broad spectrum of different pathologies, such as cardiovascular or autoimmune diseases, cancers and neurodegenerative disorders.

## 1. Introduction

MicroRNAs (miRNAs) are found in protozoa, plants and animals [1], and are known for their traditional role as post-transcriptional fine-tune regulators [2,3]; however, in the recent years, miRNAs have been investigated and associated with playing a new regulatory level: as an information medium, able to interact across the many different species of RNA molecules, establishing an elaborate dynamic balance among transcriptional products [4].

### 1.1. RNA Molecules Landscape and their Classical Roles

A variety of RNA molecules has been subjected to meticulous classifications [5] and their growing number subtypes, in particular, the class of coding and non-coding RNA with a potential regulatory effect have been the subject of in-depth studies [6,7,8], as well as being the topic of many functional annotation resources [9,10,11,12]. Beside the most famous RNA subtypes, such as coding messenger RNAs (mRNAs), transfer RNAs (tRNAs) and ribosomal RNAs (rRNAs), recent research has taken a particular interest in pseudo-genes (Ψ-genes), long non-coding RNAs (lncRNAs) and circular RNAs (circRNAs) [6]. The non-coding elements have been shown to represent one of the largest portions of transcribed molecules [12,13,14,15], and to be involved in a very broad set of biological processes [16,17,18,19], cell-fate programming [20], aging [21,22] and diseases [23].

lncRNAs cooperate in gene regulation [17,24,25], from transcriptional and post-transcriptional levels [26,27] to translational and post-translational commitments [28,29], up to epigenetic [30,31] and cell signaling modulation [32]. Ψ-genes, once considered “genomic fossils”, play a fundamental role in the regulation of their cognate genes [33] and circRNAs, far from being considered experimental artifacts, participate in transcriptional and post-transcriptional gene regulation of their parental genes through interactions with specific spliceosomal components in the nucleus [34,35].

This list includes at least one other fundamental class: microRNAs (miRNAs). MicroRNAs are a subtype of small non-coding RNA of about 20–22 nucleotides in length [36] and are produced through an elaborate biogenesis process [3,37,38], starting from transcription in the nucleus until cytoplasmic processing by the RNase III enzyme Dicer [39]. Mature miRNAs are integrated into the RNA-induced silencing complex (RISC), a multiprotein complex, and guide it to target transcripts, usually interfering with their translation and sometimes even promoting their degradation [40,41]. Target recognition is driven by a partial sequence complementarity mechanism based on a short microRNA response element (MRE) sequence found on a transcript and the “seed” sequence on the miRNA, a 6 to 8-nt sequence highly conserved across species. Interestingly, this regulation mechanism is characterized by a high complexity for distinct miRNAs are able to modulate the expression of more than one target transcript, and conversely, each transcript, harboring different MREs, may be regulated by multiple miRNAs [2,42]. Even mutations that seem negligible can significantly affect this mechanism—a change in the seed sequence may alter a specific miRNA target set and a change in an MRE can free a target from the miRNA-modulation [40,43]. In particular, it was shown that non-coding RNA (ncRNA) molecules, such as pseudo-genes, lncRNA and circRNA, represent a large reservoir of putative miRNA targets, as they too harbor MREs and can thus be bound by mature miRNAs. This specific ability of miRNAs to regulate various types of RNAs represents one of the most intriguing discoveries and provides a possible explanation of many aspects of fine-tuned post-transcriptional gene regulation [2,44].

### 1.2. Competing Endogenous RNA (ceRNA) Hypothesis

**Figure 1:**
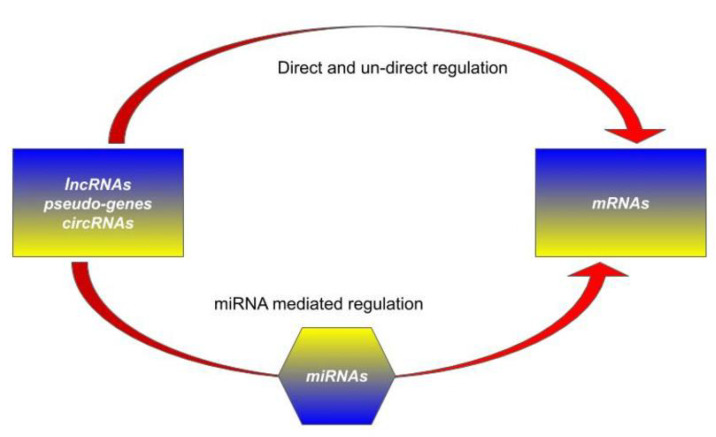
Transcription and post-transcription regulation of messenger RNAs (mRNAs) can be affected by several direct and indirect mechanisms involving circular RNAs (circRNAs), pseudo-genes (Ψ-genes) and long non-coding RNAs (lncRNAs). Some of these processes act on the transcription rate in the nucleus through the specific RNA–RNA complex, some others help the stability of mRNA molecules in the cytoplasm. Alongside this, the competing endogenous RNA (ceRNA) mechanism offers a parallel and complementary way through the same actors, protein coding and non-coding RNAs, but instead mediated by microRNAs (miRNAs).

Since miRNAs can recognize their target sites on different RNA molecules, it was suggested that miRNAs could be capable of mediating a regulatory crosstalk between the various components of the transcriptome. This miRNA regulation should be modulated by other RNA molecules, as both mRNAs and non-coding RNAs are known to be bound by miRNA and to be significantly expressed in many different biological conditions. This mechanism offers an additional post-transcriptional gene regulation mechanism and a complementary point of view for the role of the large number of transcribed, but not translated, RNAs (see Figure 1) [4].

**Figure 2:**
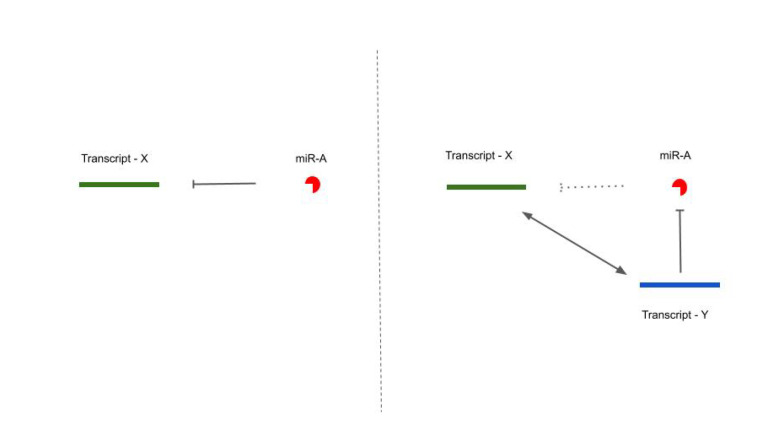
**Left panel**: A naive situation with one miRNA: miR-A, and one target: transcript-X. Transcript-X harbors miR-A microRNA response elements (MREs) and can be post-transcriptionally regulated by miR-A. **Right panel**: In the same situation, a new miR-A target is added, transcript-Y. Transcript-Y harbors itself miR-A MREs and can sponge miR-A, thus leading to a reduced post-transcriptional regulation of transcript-X by miR-A through an indirect crosstalk.

When two or more RNAs share common miRNA response elements (MREs) (mostly on their 3’ untranslated region (3’UTR), in the case of mRNAs) they can be targeted by the same microRNA(s), implying that they can cross-regulate each other indirectly because they compete to bind the same pool of sequences (see Figure 2) [4,45].

**Figure 3:**
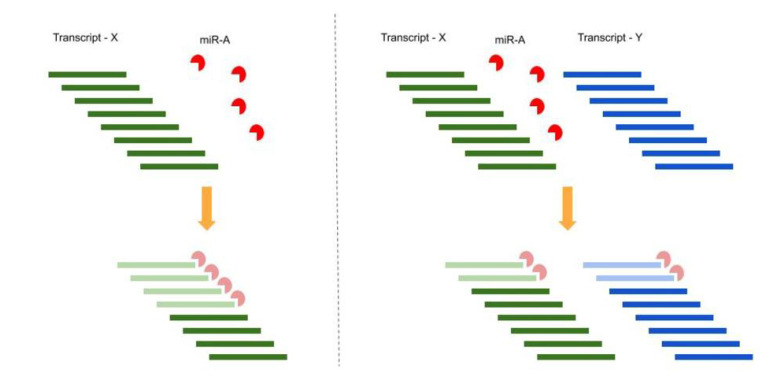
**Top left panel**: Before interaction, a naive situation with one miRNA: miR-A, and one target: transcript-X, in a 1:2 proportion ratio. Transcript-X harbors one miRNA response element (MRE) per molecule. **Bottom left panel**: After interaction, at the steady state, 50% of transcript-X molecules are under the miRNA repressive action (whether post-transcriptional degradation or translational repression) and 50% of transcript-X molecules are not affected by miR-A control. **Top right panel**: Before interaction, a new miR-A target is added: transcript-Y, in the same amount of transcript-X. Transcript-Y also has the same MRE per molecule. The new ratio between miR-A and transcript-X is 1:4. **Bottom right panel**: After interaction, at the steady state, miRNAs are shared in the same proportion between the two transcripts’ molecules. On average, 75% of transcript-X molecules are free from miRNA regulation without any changes in the transcriptional rate of transcript-X.

For instance, we can consider a very simple and naive situation composed of one miRNA: miR-A, and one target: transcript-X harboring one MRE per molecule. In a 1:2 proportion ratio at the steady state, 50% of the transcript-X molecules are not affected by miR-A control. If a new miR-A target (transcript-Y) is added, with the same MRE per molecule and in the same amount as transcript-X, the new ratio between miR-A and transcript-X will be 1:4, leading to an average of 75% of transcript-X molecules being free from miRNA regulation without any changes in the transcriptional rate of transcript-X (see Figure 3).

In general, in this new approach, transcripts could actively communicate among them, regulating their respective expression levels through a specific language in which letters are coded into the MREs. This mechanism enlarges the number of 3’UTR regulatory possibilities—they regulate the expression of the encoded proteins acting in *-cis* and could modulate the abundance of other transcripts in *-trans* by sequestering miRNA molecules. The same mechanism extends to gene regulation network relationships, adding a novel layer of indirect interactions [42,46,47,48]. The competing endogenous RNAs (ceRNAs) mechanism provides a potential explanation of some of the unexpected effects elicited by highly up- or down-regulation [4,49,50,51]. Strong down-regulation of an miRNA-modulated transcript can release a large number of miRNA molecules, which would become free to bind to other target transcripts and hyper-repress them. Conversely, overexpression of an miRNA-modulated transcript can sequester a higher number of miRNA molecules, thus de-repressing other target transcripts.

Several in silico prediction strategies were devised as a corollary of the new ceRNA logic. Transcripts acting as ceRNA should show a correlated expression trend among themselves and an anticorrelated tendency with the miRNAs they compete with [49,52,53,54]. A fundamental step is represented by the predictions of miRNA-transcript interactions. In silico models rely mostly on the analysis of seed matching sequences and thermodynamic constraints (interacting free energy and RNAs secondary structures) [55,56] whereas other techniques, like those based on CLASH (crosslinking, ligation and sequencing of hybrids) or CLIP (crosslinking and immunoprecipitation) strategies, provide new evidence of non-canonical binding sites, enlarging and better specifying the miRNA-targets landscape [57,58,59,60]. ceRNA interaction databases combine differential expression and co-expression considerations of the putative long- and short-RNA players, and provide a scoring system based on the number of shared miRNAs, reflecting the assumption—the more common miRNAs transcripts share, the stronger their reciprocal modulation [52,61,62,63,64,65]. Alongside other ceRNA bioinformatic inferring packages [66,67,68], other in vitro and in vivo experimental evidence [50,69] was produced to support this additional layer of post-transcriptional regulation; this evidence simultaneously appeared both immediately captivating and highly disputed.

Doubt was raised by considering the effective stoichiometric tolerance of RNA molecules and the extent of this mechanism in the cell life, from physiological, pathological and aberrant contexts. In this respect, different mathematical models were proposed in order to demonstrate, at least in principle, the feasibility of this mechanism [42,70,71]. The predictions offered by these mathematical models revealed that ceRNA-mediated cross-regulation depends on several factors. The absolute and relative abundance of RNA molecules, miRNAs-ceRNAs binding affinity and the number of MREs are among the most relevant. According to mathematical results, optimal conditions for ceRNA activity are reached when the number of seeds for an miR-family and its MREs are near equimolarity [42]. In such an environment, a small change in one or few transcript expression levels would greatly influence those of its or their ceRNAs by increasing or decreasing the number of free miRNA molecules.

To consider the possibility and extent of the proposed ceRNAs molecular permissive context in more depth, other biological models were paired with proper mathematical models [72,73,74,75,76]. This approach resulted in new criticisms and proposed new solutions. On the one hand, the abundance of targets seems to produce a conceptual obstacle—since each target is typically responsible only for a small fraction of the total MREs pool, it is unlikely that a variation in the expression of one of them can affect the others’ through a ceRNAs effect [72]. On the other hand, the emphasis on MREs’ hierarchical binding seems to open new perspectives: when miRNAs are expressed at a medium range and the miRNAs:targets ratio is low, we see that miRNAs are likely to bind to high-affinity sites (from 8mers to 6mers, according to TargetScan [55] seed classification). These “high-affinity” targets are shown to be more responsive to ceRNAs crosstalk and, whether the number of miRNA molecules and “high-affinity” targets verges on equimolarity, ceRNA crosstalk can be triggered by a relatively small number of additional targets [73]. It must however be stressed that non-canonical miRNA-target association sites, with their possible relevancy, function both in repression activity and competition. Previously described techniques [57,58,59,60] allow for the detection of non-canonical miRNA-target association sites even if the difficulty in knowing their precise extent only partially grants their use in mathematical models [73,74].

Alongside the first protein-coding genes ceRNA networks and lncRNA-ceRNA interactions, the growing availability of circRNAs sequences and expression data allows us to add them to the ceRNA regulatory molecules repertoire. This class of RNA has been identified since the mid-1980s, as a result of a specific type of exon scrambling, where a downstream splice donor site of an exon meets an upstream splice acceptor site [77], but it is the use of high-throughput sequencing techniques and specific alignment algorithms that have highlighted the widespread presence of these molecules in cell systems and made their systematic characterization possible [78,79]. circRNAs show cell- and tissue-specificity expression profiles, together with high expression levels, supporting their relevance in biological functions [35]. They are particularly effective as sponges because they harbor a high number of MREs [80] and exhibit an important characteristic as stable regulators—their enduring lifetime. circRNAs are less exposed to degradation driven by exonucleases because they lack a polyadenylated tail and show a median half-life 2.5 times longer than the median half-life of their linear counterparts [81]. In this respect, circRNAs are more likely to be useful as sponges and to enter into ceRNA circuitry than “linear” molecules [82].

Ever since this hypothesis was suggested, the number of papers containing references to competing endogenous RNAs is constantly growing, demonstrating that this new and indirect regulatory mechanism has made its way into the already rich panorama of biological schemes [45,83,84,85].

## 2. ceRNA and Diseases

Instances of ceRNA crosstalk were experimentally tested in a very large number of contexts and were observed in both normal and pathological backgrounds, demonstrating the wide spread proliferation of the mechanism. In particular, researchers collected evidence from normal physiology, for instance in brain architecture [86] and regeneration mechanisms [87], neuronal and muscle developmental processes [50,88], cellular differentiation [89] and reprogramming [90,91] and from the immense landscape of diseases, syndromes and disorders where highly complex gene regulation circuits are most affected by perturbations. Researchers work hard to model these regulatory networks in order to predict and understand how modifications could alter the dynamic balance among molecules, causing illness such as the onset of cancer and its progression, cardiovascular problems and neurodegenerative disorders and other pathologies, such as those relating to the immune and autoimmune response and to degenerative physical condition.

### 2.1. ceRNA and Cardiovascular Problems

Cardiovascular diseases are the leading causes of death worldwide. The aberrant balances of coding and non-coding RNA molecules are often a reflection of, or the cause of the high complexity of cardiovascular pathologies—from cardiac ischemia to cardiac fibrosis, from pathological cardiac hypertrophy to blood vessels deficiencies. Many miRNAs are related to cardiogenesis, heart development and heart normal functioning [92]: muscle-specific microRNAs (myomiRs) like miR-1 and miR-133a are involved in embryonic stem cell development and cardiac-specific muscle lineage commitment, whereas miR-208 and miR-499 collaborate to differentiate cardioblasts into cardiomyocytes and to properly specify fast and slow muscle fiber by regulating the expression of sarcomeric contractile proteins. miRNAs were proposed as potential therapeutic targets [93], but a global understanding of ncRNAs is necessary.

Some ncRNAs-miRNAs-mRNAs networks were analyzed, leading to the discovery of numerous lncRNAs functional modules in heart failure [94] and in cardiac hypertrophy (CH), involving important oncogenic and well-characterized disease-related lncRNAs, such as HOX transcript antisense intergenic RNA (HOTAIR) [95] or myocardial infarction and associated transcript (MIAT) [96,97]. Specifically in CH, the crucial role of three new characterized lncRNAs (SLC26A4-AS1, RP11-344E13.3 and MAGI1-IT) was proven [98] by combining miRNA-transcript interactions, expression data of cardiac hypertrophy from an expressly re-annotated gene expression dataset, publicly available on Gene Expression Omnibus (GEO, a public functional genomics data repository), and an analysis of the most important network topological features like the degree, betweenness and closeness. Studies on cardiomyocytes highlighted the role of miR-489 and of its target *Myd88* (myeloid differentiation primary response gene 88). miR-489 was found down-modulated in a microarray study conducted to investigate miRNA differential expression in response to angiotensin II treatment. Further, in vitro studies revealed miR-489 involvement in cardiomyocyte hypertrophy—its knockdown by antagomiRs promoted cardiomyocyte hypertrophy and its overexpression resulted in the reduction of hypertrophic responses. Among the different miR-489 target genes, *Myd88* was already involved in cardiomyocyte hypertrophy. In an experimental setting regarding angiotensin II treatment, miRNA expression changes were shown to impact the expression of the target gene and on the observable hypertrophic phenotype, revealing a functional relationship between miR-489 and *Myd88* in hypertrophy. The same angiotensin II treatment perturbation shows a time-dependent up-regulation of cardiac hypertrophy related factor (CHRF) lncRNA levels. This lncRNA is able to directly bind to miR-489 and, under this pathological condition, regulates hypertrophy by impacting on miR-489 activity and, indirectly, on *Myd88* expression (see Table 1) [99].

Interestingly, the same CHRF lncRNA was shown to act in cardiac hypertrophy through the axis miR-93-*Akt* [100]—the role of miR-93 (known to be involved in the progression of cardiac hypertrophy) was analyzed in combination with the behavior of its direct target, CHRF lncRNA. Experiments conducted in an isoproterenol induced-hypertrophy setting investigating cardiomyocytes showed the increased CHRF expression and, conversely, the decreased miR-93 expression and suggested the potential endogenous binding between miR-93 and its lncRNA target. miR-93 protein coding targets were analyzed in order to identify a possible specific gene responsible for cardiac hypertrophy. *Akt3* was selected as it was found in the overlap among the protein-coding genes putative miR-93 targets and the PI3K/Akt signaling pathway—crucial regulators in the progression of cardiac hypertrophy. The authors were able to demonstrate that the high-expression of CHRF can sponge miR-93 expression and impact cardiac hypertrophy by altering *Akt3* expression, even if pathway-specific regulatory effects remain to be further elucidated [100,101].

Another study suggests the important role of lncRNA autophagy-promoting factor (APF) in the molecular regulation of the autophagic program and myocardial infarction: in this biological context, the translation of autophagy related 7 gene (*ATG7*), involved in ischemia/reperfusion-induced myocardial injury, can be suppressed by miR-188-3p, an miRNA participating in autophagy inhibition and cell death. APF lncRNA directly binds to and competes for miR-188-3p regulating *ATG7* expression and the consequent cardiac autophagy [102]. In specific myocardial infarction mouse models and in fibrotic cardiac fibroblasts, another lncRNA was found related to cardiac dysfunctions—pro-fibrotic (PFL) lncRNA inhibits the platelet-activating factor receptor (*PTAFR*) gene by competing for miR let-7d, and leads to fibrogenesis by increasing cell viability and promoting fibroblast-myofibroblast transition [103]. The same let-7d miR represents a hub node in a specific ceRNA network based on the high-throughput RNA sequencing data of cardiac fibroblasts from neonatal mice treated with cardiac fibrosis (CF) induced by *TGF-beta1*. This miRNA is a key component of a module characterized by cardiac fibrosis-related signaling pathways, like the transforming growth factor beta (TGF-beta) signaling pathway and AMPK signaling pathway and some putative ceRNAs of it (novel-circ-0011565, novel-circ-0010678 and novel-circ-0010219), found in the same module, may have a role in determining the progression of this pathology [104]. In a similar signaling pathway, *TGF-beta1* transcript is also found to be regulated by miR-141 and, in a cardiac fibrosis context, this modulation is ceRNA-mediated by circRNA-010567 [105]; the same CF models show how several fibrosis-related genes could be under the regulatory effect of circ-ceRNA. In mouse CF models, circRNA-000203 was found to be up-regulated, it sponged miR-26b-5p and it was able to suppress the interaction of miR-26b-5p with *Col1a2* and *CTGF*, to increase expression of *Col1a2*, *Col3a1* and *alpha-SMA* genes and globally to eliminate the antifibrotic effect of miR-26b in this pathology [106]. The same genes, *alpha-SMA* and *COL3A1*, together with *COL1A1*, were found under the ceRNA regulation of circHIPK3 through the action on miR-29b-3p. When overexpressed in vitro, circHIPK3 sponged miR-29b-3p and reversed the miR-induced inhibition of cardiac fibroblasts proliferation and migration by altering the expression levels of miR-29b-3p targeting genes (*COL1A1*, *COL3A1* and *alpha-SMA*) [107].

Several other specific conditions, such as coronary artery disease and nonvalvular persistent atrial fibrillation, have been associated with circRNAs, suggesting, respectively, that circ-YOD1 is a potential biomarker [124] and that circRNA002085 and circRNA001321 show evidence of circRNA-associated ceRNA mechanisms [125].

Furthermore, circRNAs and their paired miRNAs have been associated with atherosclerosis [126,127] and myocardial infarction [128,129], cardiac hypertrophy [130,131] and diabetic cardiomyopathy [106,132], aortic aneurysm [133] and ischemic heart disease [134,135] by allowing the expansion of the interconnection of RNA molecules and pathways affected by different pathologies [136].

### 2.2. ceRNA and Neurodegenerative Disorders

The understanding of ceRNA involvement in the broad spectrum of brain-related diseases has increased dramatically in recent years.

Notably, one of the first discoveries of circRNA putative ceRNA involvement was found in neuronal tissues. Characterized in a systematic screening of circRNAs in animals [80], the circRNA CDR1as, antisense to the cerebellar degeneration-related protein 1 transcript, showed 74 MREs for the highly conserved miR-7, 63 MREs, in particular, conserved in at least one species. CDR1as was characterized by a stable and well detectable expression in the cytoplasm; in several brain regions, as in mesencephalon, this circRNA showed significant co-expression with miR-7 and, moreover, was densely bound by miRNA effector Argonaute (AGO) proteins. To further prove this post-transcriptional regulation and support this competing balance, human and mouse CDR1as circular sequences were injected into *Danio rerio* animal model embryos and the specific brain phenotype obtained was similar to the phenotype caused by miR-7 knockdown.

Later on, ceRNAs crosstalk was associated with many facets of the biological processes involved in the formation and functioning of the central nervous system, such as nerve injury repair in axon regeneration through circ-Ankib1 action on different miRNAs in Schwann cells [137] or the proper activity of human brain microvascular endothelial cells (HBMEC) where lncRNAs X-inactive specific transcript (XIST), when down-modulated, impacts the vascular endothelial growth factor (VEGF) signaling pathway by impairing hypoxia-induced angiogenesis via the miR-485-3p/*SOX7* axis [109].

lncRNAs and circRNAs are crucial in numerous neurodegenerative pathologies too [138] and evidence of RNAs balance disruption and ceRNA crosstalk was observed in Alzheimer’s and Parkinson’s disease [139,140] and Spinocerebellar Ataxia Type 7 (SCA7). In particular, several lncRNAs are known to exhibit abnormal expression in several types of cancers and in brain disorders, like lncRNA HOX transcript antisense intergenic RNA (HOTAIR) that exerts its regulatory roles in cell apoptosis by sponging miR-221 in specific Parkinson’s disease cell lines [141], or in the same context of Parkinson’s disease, like lncRNA MALAT1 (metastasis-associated lung adenocarcinoma transcript 1), through its involvement in dendritic and synapse development. An investigation into the role of MALAT1 in animal and in vitro models revealed its ceRNA function is linked to neuron apoptosis through the sponge effect on miR-124 [142]. The same miR-124 characterizes the SCA7 pathology by mediating the interaction of lnc-SCA7 and Spinocerebellar Ataxia Type 7 Protein (*Atxn7*) transcripts [110]. lnc-SCA7 is a retropseudogene highly conserved across mammals, its expression positively correlates with that of *ATXN7* in human and mouse adult tissues as well as in several central nervous system areas. miR-124 MREs found both on the 3’UTRs of mouse lnc-SCA7 and *Atxn7* suggests the possibility of competing mechanisms between the two transcripts. This ceRNA post-transcriptional regulation was proven by the observation that it is Dicer-dependent, among other evidence. lncRNA knockdown caused a significant reduction of *Atxn7* only in wild-type embryonic stem (ES) cells whereas no significant reductions were observed in Dcr-deficient ES cells. This specific ceRNA network can partially explain the selective neurodegeneration observed in SCA7. Although *ATXN7* is a ubiquitously expressed gene, miRNA-124 is most abundant in the retina and the cerebellum and lnc-SCA7 shows a stronger correlation with *ATXN7* in these same regions where the tissue-specific pathology reveals itself.

An interesting example of a ceRNA complementary mechanism, which involves lncRNAs, mRNAs and miRNAs, is found in Alzheimer’s disease (AD). The physiological expression of beta-site amyloid precursor protein cleaving enzyme 1 (*BACE1*) is fundamental for several aspects of nerve myelination [143] and synaptic functions [144]; however, elevated levels of the *BACE1* protein are linked to the formation of plaques through the generation of beta-amyloid peptides by the cleavage of amyloid precursor protein [145]. lncRNA BACE1-AS, a conserved antisense transcript overlapping *BACE1* locus exhibits a concordant expression with *BACE1* and, through the formation of a stabilizing duplex with the *BACE1* transcript, enhances the stability of *BACE1* itself [146]. *BACE1* harbor MREs for miR-485-5p, although the potential miRNA-induced translational repression is inhibited by the same transcripts duplex—BACE1-AS and miR-485-5p share a common binding site on the *BACE1* transcript and the interaction of the two transcripts masks the MRE, thus preventing miR-485-5p action. Dysregulation of this pair of miRNA and lncRNA, both found over-expressed in different regions of AD brain tissues, may induce the up-regulation of *BACE1* and consequently the onset of Alzheimer’s disease [111,112].

### 2.3. An Increasing Spectrum of Different Pathologies Involved

In vitro studies, in silico predictions and in vivo models have shown that the ceRNA mechanism is ubiquitous at a systemic level, as it could be detected in very different cellular phases and conditions. Derived from patients’ indications, specific examples on ceRNA interactions emerge from a very broad set of different syndromes.

lncRNA XIST, which is known to regulate X-chromosome inactivation by orchestrating the right gene expression on the X chromosome in female mammals [147], and miR-204, involved in arterial hypertension, diabetes, many cancer dysregulated pathways [148] but also fundamental in the development of eyes and adipogenesis [149], are found to show competing crosstalk with interferon regulatory factor 2 (*IRF2*) in lipo-polysaccharide-induced acute respiratory distress syndrome (ARDS) [114] and polycystic ovary syndrome (PCOS). The abnormal up-regulation of Prader–Willi region nonprotein coding RNA 2 (PWRN2) reduces the availability of hsa-miR-92b-3p and brings an up-regulation of hsa-miR-92b-3p direct target—transmembrane protein 120B (*TMEM120B*) protein. This up-regulation can promote adipocyte differentiation and, indirectly, cause spindle anomalies, leading to abnormal oocyte development [115].

Mutation of the *LMNA* gene can result in an accumulation in the nuclear membrane of progerin, a specific splicing isoform of *Lamin-A*, causing Hutchinson–Gilford progeria syndrome. In order to identify possible ceRNAs involved in this syndrome, several *LMNA*-predicted and validated miRNAs were used, such as miR-9—involved in neurogenesis [150] and protective against the effects of progeria [151], the tumor suppressor miR-34a [152] and miR-298 involved in Alzheimer’s disease [153]. In the top ranked list and based on the number of different shared miRNAs, key components of the RNA interference machinery, like *Dicer1*, *Argonaute* and *Drosha*, are found together with genes controlling the cell cycle, such as *TP53* and *CDKN1A*, a result that enlarges the *LMNA* interactome and suggests new interactions that could impinge on key cellular pathways [154].

ceRNAs hypothesis offers a conceptual framework to explain part of certain biological responses in syndromes characterized by large chromosomal rearrangements [155], which is also observed in certain tumor conditions. In the 5q-syndrome, bone marrow hematopoietic cells undergo the loss of the 5q31.1 band, suffering a hematological disorder that could evolve into acute myeloid leukemia. The simultaneous loss of many genes can impact the availability and abundance of a specific set of microRNAs that subsequently may alter the activity of other target transcripts belonging to other non-altered and apparently unrelated genomic regions. By miRNA–mRNA interaction in silico analysis, nine miRNA (hsa-miR-3164, hsa-miR-513a-5p, hsa-miR-30c-1-3p, hsa-miR-1254, hsa-miR-3916, hsa-miR-27a-3p, hsa-miR-27b-3p, hsa-miR-4311 and hsa-miR-665) were identified and used to pinpoint possible common target genes. To increase sensitivity and specificity, miRNAs targets were crossed with the list of transcripts found dysregulated in a differential expression analysis based on a similar syndrome in vitro setting composed by 5q- CD34+ cells compared to control CD34+ cells. The filtered gene set was particularly interesting because it contained genes not yet associated with the syndrome and two of the differentially-expressed transcripts, *GRAMD1B* and *HIPK2*, both target of all nine miRNAs, were known to be already involved in other types of leukemia.

From inflammatory responses in diabetic nephropathy [156] and vascular endothelial cells (VECs) [157] to the regulation of osteoarthritis progression [158,159] or in periodontitis [160,161], many biological processes are found involved in the class of inflammatory mechanisms.

Several lncRNAs are found involved in miRNAs sponging activities as well in degenerative mechanisms, like in for instance lumbar intervertebral disc degeneration [162] or in age-related diseases (ARDs) where lncRNAs can affect many cellular homeostasis layers [163].

Interestingly, this competing mechanism also affects immune responses, such as in liver cirrhosis [164] or in early HIV infection (EHI) gene expression regulatory networks [165] and it participates in the regulation and evolution of deceitful autoimmune diseases like rheumatoid arthritis [166,167].

### 2.4. ceRNA and Cancer

Last but not least, cancer was the first, and is one of the most studied, set of abnormalities and dysfunction-causing disease where the ceRNA mechanism has been observed. From the very beginning, every long RNA class has been associated with the ceRNA mechanism: Ψ-genes, together with their mRNA related transcript [45,69]; lncRNAs with their pervasive presence [120,168,169]; circRNAs with their large number of MREs and longer lifetime [170,171]; protein-coding RNAs themselves exhibiting coding independent functions [49].

Several new ncRNAs (both lncRNA and circRNA) are constantly highlighted through tissue- and cell-specific next generation sequencing high-throughput experiments. Predictive gene regulatory frameworks are fundamental to obtaining a high-confidence functional characterization of these new molecules and to increasing the knowledge of the precise role of ncRNA. For instance, lncRNA HOTAIR was characterized as one of the most important regulatory ncRNAs in human cells because of its oncogenic role. It is located on chromosome 12q13.13 and transcribed from an antisense strand of the HoxC gene [172]. HOTAIR was used as a prognostic biomarker for its role in the initiation and progression of different tumor types and malignancies [119,173] and characterized for its ability to regulate gene expression, specifically to repress transcripts in the *HOXD* cluster, by binding the polycomb repressive complex (PRC)2 and by recruiting (PRC)2 itself to the locus [174], or to up-regulate *SOX2* by epigenetically suppressing miR-34a and, in doing so, managing cell proliferation regulation [175]. Alongside this application, HOTAIR was associated with a competing mechanism in gallbladder cancer for its relationship with the c-Myc-activated pathway of malignancy and its negative regulation of miRNA-130a [119] and in gastric cancer for its regulation of *HER2* expression by sponging miR-331-3p [120]. ceRNA crosstalk highlighted the regulatory role of HOTAIR on the ceRNA-characterized tumor suppressor protein coding gene (*PTEN*) [45,49] but in the different biological context of cardiac hypertrophy through its inhibitor activity of miR-19 [95].

In particular, specific in vitro and in vivo models were generated to experimentally validate the involvement of Ψ-genes in ceRNA crosstalk. After the pioneering work showing that PTEN and KRAS Ψ-genes are able to affect the levels of their cognate gene [45], the role of the BRAF Ψ-gene as ceRNA was studied in an ad hoc tumorigenic system [69]. Several human cancers, including B cell lymphomas, show aberrations of BRAFP1, both at genomic and transcriptional levels; Ψ-gene BRAFP1 acts as a ceRNA with *BRAF* in human cancer cell lines, where the silencing of BRAFP1 affects MAPK signaling and cells proliferation; BRAFP1 mouse ortholog, BRAF-rs1, shows similar oncogenic activity in in vitro settings. From these considerations, mouse models, able to mimic human diffuse large B cell lymphoma, were engineered to overexpress murine BRAF Ψ-gene BRAF-rs1 and to follow its putative activity as ceRNA. Three different and independent Dox-inducible settings were planned: the overexpression of full-length Ψ-gene, its coding sequence and its 3’UTR. The oncogenic potential of Ψ-BRAF was underlined by the need to have no supplementary engineered mutations to force the onset of the phenotype and by the possibility to completely regress the tumor upon Dox withdrawal. Ψ-BRAF molecules were sensitive to miRNA activity and able to sequester specific miRNA acting on both *BRAF* and Ψ-BRAF, like miR-134, miR-543 and miR-653, leading to increased levels of *BRAF* when Ψ-gene BRAF-rs1 overexpression was activated. Though with different severity, all three engineered systems displayed a similar tumor phenotype supporting the hypothesis of an in vivo partial ceRNA regulation of *BRAF* through BRAF-rs1.

In a large portion of tumor types, from the most diffuse types, such as breast, colorectal and lung cancer, to the most rare types, such as head and neck squamous cell carcinoma or clear-cell renal cell carcinoma, Ψ-genes have been found dysregulated and their aberrant expressions, up-regulation or down-modulation, have been related to both oncogenic and tumor suppressor activities, respectively. Although Ψ-genes and Ψ-gene-derived lncRNA can affect gene expression regulation through other regulatory mechanisms, such as binding to transcription factors, several other ceRNA associations were observed involving Ψ-genes and Ψ-gene-derived lncRNA, in human cancer, sharing MREs and competing for common miRNAs with cognate or non-cognate genes [176].

*OCT4*, a key regulatory gene in the maintenance of stem cell pluripotency and proliferation, was found overexpressed in multiple human tumors too. Interestingly, in these aberrant conditions, miR-145 has been seen as a common mediator between *OCT4* and two of its Ψ-genes, OCT4-pg4 and OCT4-pg5. Specifically, in hepatocarcinogenesis, the oncogenic role of OCT4-pg4, as a ceRNA, emerges by preventing *OCT4* transcript inhibition by decoying miR-145. In particular, OCT4-pg4 is located in chromosomal region 1q22, frequently amplified in hepatocellular carcinoma and this Ψ-gene isoform, lacking the 3’UTR original region, harbors seed matches for miR-145 in the portion of sequence deriving from the coding sequence of the parental *OCT4*. Its high expression in hepatocellular carcinoma is able to sequester miR-145 molecules and, therefore, to de-repress *OCT4* leading to the coding gene aberrant high expression in this context [117]. Similarly, OCT4-pg5 is overexpressed in endometrial cancer and shows a positive correlation with OCT4 high expression in the same tumor. This Ψ-gene isoform can be directly targeted by miR-145 thanks to the MREs in the conserved 3’UTR region, and its role in miR-145-mediated endometrial carcinoma cell proliferation emerges in the regulation of *OCT4* expression, by competing for miR-145, and of PI3K/AKT-cyclin D1 signaling pathway [118].

Tumors continue to highlight many new molecular mutations and cellular defects that affect complex transcriptional and post-transcriptional balance [155,177] in almost all tissue-specific gene regulation networks [178,179,180,181,182,183,184]. International consortia efforts, such as The Cancer Genome Atlas (TCGA) [185], represent a huge step forward, and have collected and publicly shared the data of transcripts and protein expression, sequences and genomic variants and other *omics* data. Altogether, these efforts have made possible the transition from highly specific and context-dependent ncRNA–miRNA–mRNA subsets to wide-ranging systems of biological studies characterized by a much more general, complete and detailed set of regulatory networks [186,187,188].

## 3. Conclusions

This review traces a brief summary of the evolution of the ceRNA hypothesis and how the scientific community has expanded its range of application. The ceRNA mechanism was found to operate in a very broad set of biological contexts and almost all families of RNA molecules can participate in these regulatory strategies.

Together with some experimental evidence in in vivo models, the vast majority of ceRNA crosstalk is still predicted through in silico strategies and are shown to be effective in in vitro settings. Bioinformatics predictions demand caution in the acceptance of these suggestions [114] deriving from indirect biological evidence and statistical methods, but nevertheless, they demonstrate that molecules can participate in this regulatory mechanism and, at the same time, offer a large number of relationships that can be tested experimentally. The same miRNA identifiers used reflect the usage of mixed experimental strategies, which require bioinformatics analysis and studies on animal models and human cell lines. miRNAs are often indicated through their family name without specifying the species prefix, reflecting their involvement in relationships that play an important role in different species, demonstrating further the broad conservation of the action of the miRNAs themselves.

In particular, the importance of the ceRNA mechanism is related to its capacity to offer new indications for diagnostic and prognostic putative biomarkers [94,189], targets for drugs such as in hypertrophic scars treatment [190] and predictions for therapeutic strategies, such as in the cell proliferation of complex mechanisms in regenerating livers [87,178], or as in the broad spectrum of tumors [191,192,193,194] or still as in the pathogenesis of early HIV infection and its related antiviral therapy [165].

Efforts spent in modeling ceRNA interactions and in exploring their most permissive molecular environments [195] had the advantage of having contributed to the study and knowledge of the molecules stoichiometry balance inside the cells and can further improve precision medicine associated with the other most well known complex regulatory circuits (proteomics, metabolomics and more general *omics*) [196,197].

At the same time, the organization of all the information already available, coming from thousands of studies and spanning protein and RNA expression and co-expression networks, miRNAs target prediction information, transcription factors activity, epigenetics and genomic topological knowledge should be organized in order to allow for an easy expansion and integration with future studies and to make immediately accessible the wealth of details and particularities that biological interconnections offer at the level of different cells, tissues and organs contexts, stages of development and aging. As well as dynamic and multipartite network approaches should be adopted to display information and to browse across interactors, cellular contexts and diseases.

**Figure 4:**
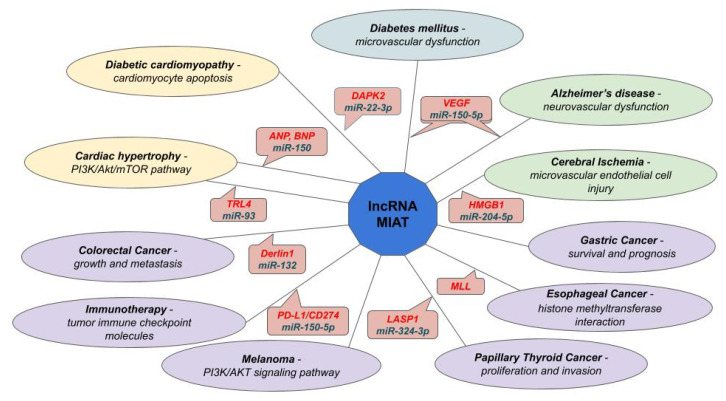
Partial representation of regulatory interaction of lnc-myocardial infarction and associated transcript (MIAT): in ellipses, diseases are reported in bold and dysfunctions in italics; in comics, interacting ceRNAs, or influenced transcripts, are reported in italics red and miRNAs in italics blue. Different colors of ellipses are linked to different types of pathologies.

As a small and non-exhaustive example, lncRNA-myocardial infarction and associated transcript (MIAT) was characterized in a large variety of conditions [198] (see Figure 4). As already mentioned when discussing cardiac hypertrophy, MIAT has been found acting on at least two distinct miRNAs in this abnormal enlargement and thickening of the heart muscle. miR-150, an important miRNA involved in cardiac and cardiomyocyte hypertrophy, affects the development of cardiac hypertrophy as a downstream effector of MIAT [96]. miR-93 differential abundance, mediated by lncRNA-MIAT, influences the expression of target genes, as the highly conserved Toll-like receptor 4 (*TLR4*). In particular, MIAT knockdown can enhance miR-93 and inactivate the PI3K/Akt/mTOR pathway via regulating the *TLR4* in angiotensin II-induced cardiac hypertrophy [97]. MIAT works as a ceRNA in pathological angiogenesis related to diabetes mellitus microvascular dysfunction, by competing with vascular endothelial growth factor (*VEGF*) for miR-150-5p [116] and, following the same pathway, in neurovascular dysfunction by inducing progressive neuronal loss and Alzheimer’s disease [113]. After cerebral ischemia, lncRNA-MIAT has proved to regulate the expression of *HMGB1* (high-mobility group box 1) in cerebral microvascular endothelial cell (CMEC) injury by competing for miR-204-5p [84]. The same lncRNA has been associated with diabetic cardiomyopathy (DCM) for it affects the expression of death-associated protein kinase 2 (*DAPK2*) by sponging miR-22-3p: the resulting up-regulation of *DAPK2* itself leads to cardiomyocyte apoptosis in DCM [108]. Its involvements in cancer are emerging too: in melanoma by acting on key master regulators of the signaling pathway [199]; in papillary thyroid cancer by sponging hsa-miR-324-3p and up-regulating LIM and SH3 domain protein 1 (*LASP1*) [122]; in colorectal cancer by regulating the miR-132/Derlin-1 pathway [121]. New observations have emerged in the control of immune checkpoint molecules: through hsa-miR-150-5p sponge interaction, lncRNA-MIAT, together with HLA complex P5 (*HCP5*), has been associated with the up-regulated expression of *PD-L1/CD274*, suggesting new involvements in the field of tumor immunity and immunotherapy [123]. Independently from the ceRNA mechanism, the same lnc-RNA MIAT molecules were characterized as oncogenic in esophageal cancer, as they promote cell invasion and migration by interacting with histone methyltransferase mixed-lineage leukemia (*MLL*) proteins [200] and, moreover, in gastric cancer, they have been linked to the prognosis and survival predictions: for instance, high MIAT level in serum exosomal characterizes patients as more prone to develop gastric cancer and its up-regulation is associated with shorter survival periods and represents an independent prognostic factor for gastric cancer [201].

ceRNA crosstalk is widespread in many contexts and the integration of this mechanism with all the other layers of gene regulation will guarantee new ways of increasing the understanding of molecular and cellular mechanisms and to intervene in increasingly punctual and specific ways in medicine.

## Figures and Tables

**Table 1:** Table summarizing the miRNA-ceRNAs networks discussed in the review. Table fields are: mRNA, protein coding genes name; ncRNA, non-coding RNAs class and name; miRNA, microRNA involved; Disease; Reference. ^(a)^ HBMEC, Human Brain Microvascular Endothelial Cells; ^(b)^ SCA7, Spinocerebellar Ataxia Type 7; ^(c)^ ARDS, Acute Respiratory Distress Syndrome; ^(d)^ PCOS, Polycystic Ovary Syndrome; ^(e)^ CMEC, Cerebral Microvascular Endothelial Cell Injury.

mRNA	ncRNA	miRNA	Disease	Reference
*Myd88*	lncRNA—CHRF	miR-489	Cardiac Hyperthrophy	[99]
*Akt3*	lncRNA—CHRF	miR-93	Cardiac Hyperthrophy	[100,101]
*TRL4*	lncRNA—MIAT	miR-93	Cardiac Hyperthrophy	[97]
*PTEN*	lncRNA—HOTAIR	miR-19	Cardiac Hyperthrophy	[95]
*ATG7*	lncRNA—APF	miR-188-3p	Cardiac Autophagy	[102]
*PTAFR*	lncRNA—PFL	let-7d	Cardiac Fibrosis	[103]
TGF-beta pathway	circ-0011565	let-7d	Cardiac Fibrosis	[104]
TGF-beta pathway	circ-0010678	let-7d	Cardiac Fibrosis	[104]
TGF-beta pathway	circ-0010219	let-7d	Cardiac Fibrosis	[104]
*TGF-beta1*	circRNA-010567	miR-141	Cardiac Fibrosis	[105]
*Col1a2*	circRNA-000203	miR-26b-5p	Cardiac Fibrosis	[106]
*CTGF*	circRNA-000203	miR-26b-5p	Cardiac Fibrosis	[106]
*COL1A1*	circHIPK3	miR-29b-3p	Cardiac Fibrosis	[107]
*COL1A3*	circHIPK3	miR-29b-3p	Cardiac Fibrosis	[107]
*Alpha-SMA*	circHIPK3	miR-29b-3p	Cardiac Fibrosis	[107]
*DAPK2*	lncRNA—MIAT	miR-22-3p	Diabetic Cardiomyopathy	[108]
*SOX7*	lncRNA—XIST	miR-485-3p	HBMEC ^(a)^	[109]
*Atxn7*	retro-Ψ-gene—lnc-SCA7	miR-124	SCA7 ^(b)^	[110]
*BACE1*	lncRNA—BACE1-AS	miR-485-5p	Alzheimer’s Disease	[111,112]
*VEGF*	lncRNA—MIAT	miR-150-5p	Alzheimer’s Disease	[113]
*HMGB1*	lncRNA—MIAT	miR-204-5p	CMEC ^(e)^	[84]
*IRF2*	lncRNA—XIST	miR-204	ARDS ^(c)^	[114]
*TMEM120B*	lncRNA—PWRN2	miR-92b-3p	PCOS ^(d)^	[115]
*VEGF*	lncRNA—MIAT	miR-150-5p	Diabetes Mellitus	[116]
*BRAF*	Ψ-gene—Ψ-BRAF	miR-134; miR-543; miR-653	Diffuse Large B Cell Lymphoma	[69]
*OCT4*	Ψ-gene—OCT4-pg4	miR-145	Hepatocellular Carcinoma	[117]
*OCT4*	Ψ-gene—OCT4-pg5	miR-145	Endometrial Carcinoma	[118]
C-Myc pathway	lncRNA—HOTAIR	miR-130a	Gallbladder Cancer	[119]
*HER2*	lncRNA—HOTAIR	miR-331-3p	Gastric Cancer	[120]
*Derlin1*	lncRNA—MIAT	miR-132	Colorectal Cancer	[121]
*LASP1*	lncRNA—MIAT	miR-324-3p	Papillary Thyroid Cancer	[122]
*PD-L1/CD274*	lncRNA—MIAT	miR-150-5p	Immunotherapy Involvement	[123]
